# Survival in Early Phase Immuno-Oncology Trials: Development and Validation of a Prognostic Index

**DOI:** 10.1093/jncics/pkz071

**Published:** 2019-09-19

**Authors:** Daphne Day, Christina Guo, Yada Kanjanapan, Ben Tran, Anna Spreafico, Anthony M Joshua, Lisa Wang, Albiruni R Abdul Razak, Natasha B Leighl, Aaron R Hansen, Marcus O Butler, Lillian L Siu, Jayesh Desai, Philippe L Bedard

**Affiliations:** 1 Division of Medical Oncology and Hematology, Princess Margaret Cancer Centre, Toronto, Canada; 2 Department of Medicine, University of Toronto, Toronto, Canada; 3 Department of Medical Oncology, Peter MacCallum Cancer Centre, Parkville, Melbourne, Australia

## Abstract

**Background:**

Immuno-oncology (IO) is rapidly evolving in early drug development. We aimed to develop and prospectively validate a prognostic index for patients treated in IO phase I trials to assist with patient selection.

**Methods:**

The development cohort included 192 advanced solid tumor patients treated in 13 IO phase I trials, targeting immune checkpoint and/or co-stimulatory molecules. A prognostic scoring system was developed from multivariate survival analysis of 10 clinical factors, and subsequently validated in two independent validation cohorts (n = 152 and n = 80).

**Results:**

In the development cohort, median age was 57.5 years (range = 20.4–84.8 years). Median progression-free survival and overall survival (OS) were 13.4 and 73.6 weeks, respectively, 90-day mortality was 16%, and overall response rate was 20%. In multivariate analysis, Eastern Cooperative Oncology Group performance status greater than or equal to 1 (hazard ratio [HR] = 3.2, 95% confidence interval [CI] = 1.8 to 5.7; *P* < .001), number of metastatic sites greater than 2 (HR = 2.0, 95% CI = 1.3 to 3.1; *P* = .003), and albumin less than the lower limit of normal (HR = 1.8, 95% CI = 1.2 to 2.7; *P* = .007) were independent prognostic factors; comprising the Princess Margaret Immuno-oncology Prognostic Index (PM-IPI). Patients with a score of 2–3 compared with patients with a score of 0–1 had shorter OS (HR = 3.4, 95% CI = 1.9 to 6.1; *P* < .001), progression-free survival (HR = 2.3, 95% CI = 1.7 to 3.2; *P* < .001), higher 90-day mortality (odds ratio = 8.1, 95% CI = 3.0 to 35.4; *P* < .001), and lower overall response rate (odds ratio = 0.4, 95% CI = 0.2 to 0.8; *P* = .019). The PM-IPI retained prognostic ability in both validation cohorts and performed better than previously published phase I prognostic scores for predicting OS in all three cohorts.

**Conclusions:**

The PM-IPI is a validated prognostic score for patients treated in phase I IO trials and may aid in improving patient selection.

Immuno-oncology (IO) therapies such as monoclonal antibodies targeting immune regulatory checkpoints, cytotoxic T-lymphocyte-associated antigen-4 (CTLA-4), programmed cell death protein-1 (PD-1), and PD-1 ligand (PD-L1) have transformed drug development in oncology. These IO therapies induce antitumor immunity and produce durable responses in patients with advanced malignancies, with approved indications in multiple tumor types (1–14). However, the majority of patients treated with IO therapies do not benefit, underscoring the complexities of the tumor-immune system relationship. Although multidrug approaches such as IO-IO or IO-cytotoxic therapy combinations may enhance treatment efficacy, there may be increased toxicities (15–18). At present, there is no precise biomarker-based approach to reliably predict for IO treatment response or resistance.

There are an unprecedented number of IO agents in early drug development, such as novel checkpoint inhibitors, co-stimulatory agonists, adoptive T-cell therapy, and vaccines, alone or in combination. A recent review reported that there are currently more than 900 IO agents in clinical development and more than 3000 active clinical trials (19). Phase I trials are the first clinical studies to evaluate the safety and efficacy of novel therapies. Because phase I trials typically involve patients with advanced refractory malignancies with short life expectancies, the appropriate selection of patients who will survive long enough is critical to evaluate the causality of adverse events and preliminarily assess the therapeutic impact of novel treatments.

Various prognostic scoring systems for patients treated in phase I oncology trials have been published using clinical parameters that are independent predictors of overall survival (OS) and/or 90-day mortality (90DM) in multivariate analysis (MVA) (Table 1) (20–28). Of these, the Royal Marsden Index, which incorporates serum albumin, lactate dehydrogenase (LDH), and number of metastatic sites (20), has been independently validated (27,29). The majority of these prognostic scores were developed in patients treated in cytotoxic and molecularly targeted phase I trials. IO therapies have distinct mechanisms of action, response patterns, and toxicities compared with cytotoxic and molecularly targeted agents. Moreover, the design and conduct of phase I trials have rapidly evolved since the publication of these prognostic scores, with larger trials that include multiple disease- and/or biomarker-enriched “basket” cohorts at the maximum tolerated dose now routinely used to evaluate IO therapies. More recently, the Gustave Roussy Institute and MD Anderson Cancer Center have both examined prognostic factors in phase I trials of immune checkpoint inhibitors and identified three and seven baseline factors, respectively, as independent predictors of OS (Table 1) (30,31).

**Table 1.** Selected previously published phase I prognostic scores*

RMI (20)	PMHI (21)	GRIm-Score (30)	MDA-ICI (31)
Cytotoxics and molecularly targeted agents		Immune checkpoint inhibitor therapy	
Albumin <35 g/L	Albumin <35 g/L	Albumin <35 g/L	Age >52 years
>2 metastatic sites	>2 metastatic sites	NLR >6	ECOG PS >1
LDH >ULN	ECOG PS >0	LDH >ULN	LDH >0.75 × ULN
			Platelet >300 × 10^3^/μL
			ANC >4.9 × 10^3^/μL
			ALC <1.8 × 10^3^/μL
			Liver metastases

*ALC = absolute lymphocyte count; ANC = absolute neutrophil count; ECOG PS = Eastern Cooperative Oncology Group performance status; GRIm-score = Gustave Roussy Immune Score; LDH = lactate dehydrogenase; MDA-ICI = MD Anderson Immune Checkpoint Inhibitor; NLR = neutrophil-lymphocyte ratio; PMHI = Princess Margaret Hospital Index; RMI = Royal Marsden Index; ULN = upper limit of normal.

We evaluated clinical characteristics and outcomes of patients treated in IO phase I trials to develop a simple, objective, and reproducible prognostic score: the Princess Margaret Immuno-oncology Prognostic Index (PM-IPI). The PM-IPI was subsequently prospectively validated in two independent cohorts.

## Methods

We identified consecutive advanced solid tumor patients treated in phase I IO trials in the Princess Margaret Early Drug Development Program between August 2012 and August 2015 from the institutional electronic database for the development cohort. A study was included if at least one of the investigational agents was an immune checkpoint inhibitor or co-stimulatory agonist. Vaccine, cytokine and T-cell therapies were not included.

We recorded and analyzed the following 10 clinical and laboratory variables at baseline, defined as within 2 weeks of trial treatment commencement: Eastern Cooperative Oncology Group (ECOG) performance status, age, number of prior systemic treatments, number of metastatic sites, serum LDH, albumin, and sodium, hemoglobin, platelet count, and neutrophil-to-lymphocyte ratio (NLR). The baseline variables were selected based on previously published prognostic scores or were identified from the literature and hypothesized to be potentially clinically relevant. Data collection also included treatment response and survival from review of patient charts, clinical research records, and cancer registries. Response evaluations were assessed by trained radiologists based on Response Evaluation Criteria in Solid Tumors (RECIST) Version 1.1, Immune-related Response Criteria, or Immune RECIST depending on the specific trial criterion used.

To validate our results, we analyzed the characteristics and outcomes of consecutive advanced solid tumor patients treated in phase I IO studies in the Princess Margaret Early Drug Development Program from September 2015 to August 2016 (validation cohort A, excluding patients from the development cohort) and in the Peter MacCallum Cancer Centre Early Drug Development Group from November 2015 to March 2018 (validation cohort B). Ethics approvals were obtained from local institutional review boards for data collection.

### Statistical Methods

The primary endpoint was OS, defined as the time from the commencement of trial treatment to death due to any cause. All patients who were alive at the time of last follow-up were censored. All variables were examined in univariate analysis as predictors of OS using the Cox proportional hazards model and 90DM using logistic regression. Martingale residuals were assessed to verify the proportionality assumption. Continuous variables were categorized based on a cutoff value that gave the greatest separation in OS. Variables with *P* values no more than .10 (two-sided) level in univariate analysis were included in the MVA logistic regression model. In MVA, only variables with *P* values below .05 (two-sided) were considered statistically significant. The final prognostic factors were incorporated into a scoring system to build the PM-IPI.

For data validation, the assumptions used for sample size analysis were based on the results from the development cohort, including the overall death rate and the three significant clinical parameters identified in MVA. To test the performance of the PM-IPI and previously reported prognostic scores, patients were subcategorized into groups according to the prognostic scores. OS was estimated using the Kaplan-Meier method, and comparisons were made using the log-rank test. The concordance index method was used to rank scores according to their capacity to discriminate patients according to OS and progression-free survival (PFS), with a value of 0.5 having no discriminative ability and a value of 1 having perfect discriminative ability. The receiver operating characteristic curve method was used to measure the discrimination of 90DM and overall response rate (ORR). Statistical analysis was performed using SAS software (SAS institute, Cary, NC).

## Results

### Patient Characteristics and Outcomes in the Development Cohort

We identified 192 patients treated in 13 phase I IO trials. Baseline characteristics are shown in Table 2. Median age was 57.5 years (range = 20.4–84.8) and 56% (n = 107) of patients were male. The most common tumor types included melanoma (27%), lung (21%), urological (11%), head and neck (10%), and gastrointestinal (9%) cancers. Of the patients, 81% (n = 156) had at least one prior systemic therapy, including 15% (n = 28) who were treated in a prior phase I trial. Thirteen percent (n = 25) of patients had prior IO therapy including anti-CTLA-4 antibodies (10%, n = 20) and cytokines (4%, n = 8). All were naïve to anti-PD-1/PD-L1 therapy. The majority (88%, n = 169) of patients received single-agent IO treatment on trial. Trial treatment characteristics are shown in Supplementary Table 1 (available online). After median follow-up of 62.0 weeks, there were 135 deaths. Median PFS and OS were 13.4 (95% confidence interval [CI] = 11.9 to 17.9) and 73.6 (95% CI = 44.9 to 93.7) weeks, respectively, and 90DM was 16%. ORR was 20%. A further 27 (14%) patients achieved stable disease for greater than 6 months. Partial and complete response compared with stable disease and progressive disease were associated with OS (*P* < .001). Following IO trial treatment, 47% (n = 96) of patients went on to receive other systemic therapies, including another phase I trial in 12% (n = 23).

**Table 2.** Baseline patient characteristics

Patient characteristic	Number of patients (%) or median (range)		
	Development cohort	Validation cohort A	Validation cohort B*
	N = 192	N = 152	N = 80
Sex			
Male	107 (56%)	80 (53%)	42 (53%)
Female	85 (44%)	72 (47%)	38 (48%)
Age, y	57.5 (20.4–84.8)	60.0 (20.0–84.8)	61.6 (19.0–82.0)
ECOG performance status			
0	76 (40%)	43 (28%)	28 (35%)
1	116 (60%)	109 (72%)	51 (64%)
≥2	0 (0%)	0 (0%)	1 (1%)
Primary tumor site			
Melanoma	52 (27%)	9 (6%)	9 (11%)
Thoracic	41 (21%)	8 (5%)	1 (1%)
Genitourinary	22 (11%)	15 (10%)	16 (20%)
Head and neck	20 (10%)	23 (15%)	8 (10%)
Sarcoma	14 (7%)	9 (6%)	6 (8%)
Gynecologic	13 (7%)	25 (16%)	14 (18%)
UGI/biliary	11 (6%)	19 (13%)	14 (18%)
Breast	8 (4%)	8 (5%)	1 (1%)
Colorectal	5 (3%)	16 (11%)	8 (10%)
Other	6 (3%)	20 (13%)	3 (4%)
Time from diagnosis of advanced disease to trial treatment (wks)	67.3 (3.9–498.4)	68.4 (1.6–621.9)	98.7 (2.9–739.0)
Number of prior systemic therapies	2 (0–8)	1 (0–7)	1 (0–9)
<2	81 (42%)	86 (57%)	42 (53%)
≥2	111 (58%)	66 (43%)	38 (48%)
Prior IO therapy†	25 (13%)	9 (6%)	3 (4%)
Anti-CTLA-4 antibody	20 (10%)	5 (3%)	0 (0%)
Anti-PD-1/PD-L1 antibody	0 (0%)	5 (3%)	3 (4%)
Cytokine therapy	8 (4%)	1 (1%)	0 (0%)
Other IO therapy	2 (1%)	1 (1%)	0 (0%)
Number of metastatic sites	3 (0–7)	3 (1–7)	1 (0–5)
≤2	86 (45%)	75 (49%)	58 (73%)
>2	106 (55%)	77 (51%)	22 (28%)
Sites of metastasis			
Lung	123 (64%)	91 (60%)	35 (44%)
Liver	74 (39%)	62 (41%)	26 (33%)
Bone	52 (27%)	39 (26%)	24 (30%)
Brain	23 (12%)	5 (3%)	3 (4%)
Hemoglobin g/L‡	123 (81–157)	124 (87–165)	117 (82–161)
<LLN	125 (65%)	100 (66%)	41 (51%)
≥LLN	67 (35%)	52 (34%)	39 (49%)
Neutrophil-to-lymphocyte ratio	4.5 (0.8–39.5)	4.4 (0.5–12.1)	3.85 (1.19–20.58)
≤4	87 (45%)	60 (39%)	44 (55%)
>4	105 (55%)	92 (61%)	36 (45%)
Platelets × 10^9^/L‡	243 (104–812)	245 (100–626)	243 (82–545)
≤ULN	178 (93%)	143 (94%)	75 (94%)
>ULN	14 (7%)	9 (6%)	5 (6%)
Sodium mmol/L‡	138 (127–149)	138 (130–146)	138 (125–143)
<LLN	31 (16%)	15 (10%)	10 (13%)
≥LLN	161 (84%)	137 (90%)	70 (88%)
LDH U/L‡	257 (130–6068)	265 (143–2917)	238 (100–608)
≤ULN	50 (26%)	48 (32%)	35 (56%)*
>ULN	142 (74%)	104 (68%)	27 (44%)*
Albumin g/L‡	40 (26–45)	40 (26–48)	39 (28–59)
<LLN	63 (33%)	34 (22%)	11 (14%)
≥LLN	129 (67%)	118 (78%)	69 (86%)

* Sixty-two of 80 patients in validation cohort B had LDH measured at baseline. CTLA-4 = cytotoxic T-lymphocyte-associated antigen-4; ECOG = Eastern Cooperative Oncology Group; IO = immuno-oncology; LDH = lactate dehydrogenase; LLN = lower limit of normal; PD-1 = programmed cell death protein-1; PD-L1 = programmed cell death ligand-1; UGI = upper gastrointestinal; ULN = upper limit of normal.

† Some patients had more than one prior IO therapy.

‡ Reference ranges: Princess Margaret Cancer Centre: hemoglobin, male 140–180 g/L, female 120–160 g/L; platelets 150–400 × 10^9^/L; sodium 135–145 mmol/L; LDH 125–220 U/L; albumin 38–50 g/L. Peter MacCallum Cancer Centre: hemoglobin 115–155 g/L; platelets 150–400 × 10^9^/L; sodium 135–145 mmol/L; LDH 120–250 U/L; albumin 35–50 g/L.

### Development of the PM-IPI

**Figure 1.**
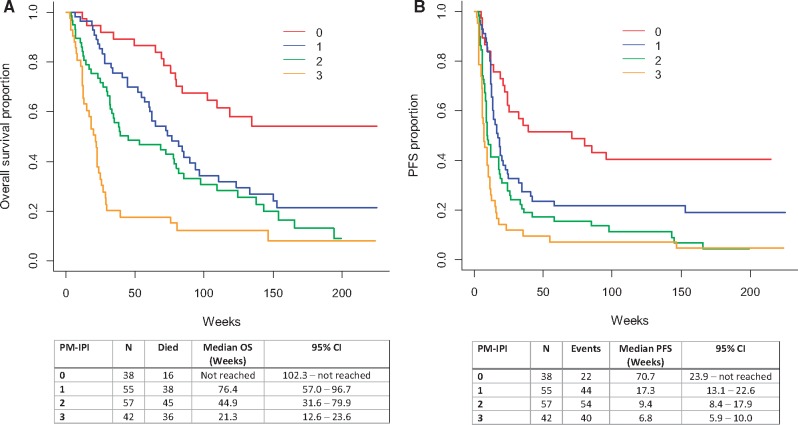
Kaplan-Meier curves for overall survival **(A)** and progression-free survival **(B)** in the development cohort based on the Princess Margaret Immuno-oncology Prognostic Index (PM-IPI). CI = confidence interval; OS = overall survival; PFS = progression-free survival.

Factors that were associated with shorter OS in univariate analysis are shown in Table 3. Age, number of prior systemic therapies, hemoglobin, and serum sodium level were not prognostic of survival in this patient cohort. In MVA, ECOG performance status greater than or equal to 1 (hazard ratio [HR] = 3.2, 95% CI = 1.8 to 5.7; *P* < .001), number of metastatic sites greater than 2 (HR = 2.0, 95% CI = 1.3 to 3.1; *P* = .003), and albumin less than the lower limit of normal (HR = 1.8, 95% CI = 1.2 to 2.7; *P* = .007) were independent prognostic factors. Each of these three prognostic factors was allocated one point, comprising the PM-IPI. Patients with a score of 2–3 compared with patients with a score of 0–1 had shorter OS (HR = 3.4, 95% CI = 1.9 to 6.1; *P* < .001), PFS (HR = 2.3, 95% CI = 1.7 to 3.2; *P* < .001), higher 90DM (OR = 8.1, 95% CI = 3.0 to 35.4; *P* < .001), and lower ORR (OR = 0.4, 95% CI = 0.2 to 0.8; *P*= .019). The median OS for patients with a PM-IPI of 0, 1, 2, or 3 was not reached (95% CI = 102.3 to not reached), 76.4 (95% CI = 57.0 to 96.7), 44.9 (95% CI = 31.6 to 79.9), and 21.3 weeks (95% CI = 12.6 to 23.6), respectively (Figure 1A).

**Table 3.** Prognostic parameters of overall survival on univariate and multivariate analysis in the development cohort*

Variable	Univariate model		Multivariable model	
	HR (95% CI)	*P*	HR (95% CI)	*P*
ECOG ≥1	4.34 (2.50 to 7.55)	<.001	3.20 (1.80 to 5.70)	<.001
Albumin <38 g/L	2.63 (1.75 to 3.94)	<.001	1.79 (1.17 to 2.72)	.007
Number of metastatic sites >2	2.41 (1.55 to 3.73)	<.001	1.96 (1.26 to 3.05)	.003
LDH >220 U/L	1.99 (1.18 to 3.37)	.01	—	—
Hemoglobin <120 g/L female or <140 g/L male	0.76 (0.49 to 1.17)	.21	—	—
Sodium <135 mmol/L	1.55 (0.93 to 2.60)	.09	—	—
Platelets >400 × 10^9^/L	3.09 (1.62 to 5.87)	<.001	—	—
Age (continuous variable)	1.00 (0.98 to 1.01)	.72	—	—
Number of prior systemic therapies (continuous variable)	1.10 (0.98 to 1.24)	.01	—	—
Neutrophil-to-lymphocyte ratio (continuous variable)	1.07 (1.04 to 1.10)	<.001	—	—

* CI = confidence interval; ECOG = Eastern Cooperative Group performance status; HR = hazard ratio; LDH = lactate dehydrogenase.

### Validation Cohorts

Validation cohort A and validation cohort B included 152 and 80 patients, respectively. Baseline patient characteristics and trial treatment characteristics are shown in Table 2 and Supplementary Tables 2 and 3 (available online). In validation cohort A, at a median follow-up of 33.0 weeks, median PFS was 9.0 weeks (95% CI = 8.0 to 11.6), and median OS was 39.7 weeks (95% CI = 34.4 to not reached). 90DM was 14%, and ORR was 7%. In validation cohort B, at a median follow-up of 69.1 weeks, median PFS was 14.4 weeks (95% CI = 8.3 to 27.3), and OS was 83.0 weeks (95% CI = 58.9 to not reached). 90DM was 19%, and ORR was 19%.

In MVA of the validation cohorts, ECOG performance status greater than or equal to 1, albumin less than the lower limit of normal, and number of metastatic sites greater than 2 were independently associated with worse OS. In validation cohort A, patients with a PM-IPI score of 2–3 compared with 0–1 had shorter OS (HR = 3.3, 95% CI = 1.7 to 6.2; *P* < .0001), PFS (HR = 1.7, 95% CI = 1.2 to 2.4; *P* = .005), higher 90DM (OR = 12.2, 95% CI = 1.6 to 100.4; *P* = .019), and a trend toward lower ORR (OR = 0.4, 95% CI = 0.1 to 1.5; *P* = .15). In validation cohort B, patients with a PM-IPI score of 2–3 compared with 0–1 had shorter OS (HR = 4.6, 95% CI = 2.3 to 9.2; *P* < .001), PFS (HR = 2.5, 95% CI = 1.5 to 4.1; *P* < .001), lower ORR (OR = 0.12, 95% CI = 0.02 to 1.0; *P* = .05), and a trend toward higher 90DM (OR = 4.4, 95% CI = 0.9 to 20.1; *P* = .06).

As shown in Table 4, the predictive discriminative ability of the PM-IPI was fair to good for OS (0.68–0.71), PFS (0.57–0.66), 90DM (0.70–0.80), and ORR (0.64) in all three cohorts. Additionally, the prognostic performance of PM-IPI was superior to other previously published phase I prognostic scores for OS (Table 5). Supplementary Figures 1 and 2 (available online) show the Kaplan-Meier plots for OS and PFS stratified by the PM-IPI score for both validation cohorts.

**Table 4.** Prognostic performance of the Princess Margaret Immuno-oncology Prognostic Index in the development and validation cohorts*

Outcome	Development cohort	Validation cohort A	Validation cohort B
OS, c-index	0.71	0.69	0.68
PFS, c-index	0.66	0.57	0.61
90DM, AUC	0.75	0.80	0.70
ORR, AUC	0.64	0.64	0.64

* 0.5 = no discriminative ability; 1 = perfect discriminative ability. 90DM = 90-day mortality; AUC = area under the curve; c-index = concordance index; ORR = overall response rate; OS = overall survival; PFS = progression-free survival; PM-IPI = Princess Margaret Immuno-oncology Prognostic Index.

**Table 5.** Comparative prognostic performance of the Princess Margaret Immuno-oncology Prognostic Index for overall survival (C-index) in the development and validation cohorts compared with previously published prognostic scores*

Prognostic index	Development cohort	Validation cohort A	Validation cohort B
PM-IPI	0.71	0.69	0.68
RMI	0.65	0.63	0.51
GRIm-Score	0.64	0.63	0.63
MDA-ICI	0.61	0.62	0.60

* 0.5 = no discriminative ability; 1= perfect discriminative ability. GRIm-score = Gustave Roussy Immune Score; MDA-ICI = MD Anderson Immune Checkpoint Inhibitor; PM-IPI = Princess Margaret Immuno-oncology Prognostic Index; RMI = Royal Marsden Index.

## Discussion

In this study, the PM-IPI was developed and independently validated, comprising three prognostic factors for OS in patients treated in phase I IO trials including ECOG performance status, number of metastatic sites, and albumin. These three factors are routinely evaluated in the clinical trial setting, making the PM-IPI easily applicable at the point of care. In all three cohorts, the prognostic performance of PM-IPI was superior to that of previously published phase I prognostic scores including the Royal Marsden Index and IO trial-specific scores, the Gustave Roussy Immune Score, and the MD Anderson Immune Checkpoint Inhibitor score for OS (Table 5). Notably, 31–52% of patients enrolled in IO trials across three independent cohorts had at least two adverse prognostic features (PM-IPI 2 or 3), demonstrating that early phase investigators frequently enroll patients with poor expected survival.

Consistent with previous reports in advanced cancer in clinical trial and nontrial populations (21, 25–29, 31–33), ECOG performance status has been found to be prognostic for survival. Performance status reflects the global fitness and functional capacity of patients. It is frequently assessed in cancer care and is a key consideration in clinical decision-making, including determining clinical trial eligibility. Similarly, albumin, as a marker of nutrition and general health, has been reported to be a prognostic marker in several previously published prognostic scores (20, 21, 24, 25, 30, 34). The number of metastatic sites may reflect overall tumor burden and has been observed to be associated with outcome in other phase I series (21, 25, 26). Emerging data in melanoma suggest that PD-1 blockade may be more effective when tumor burden is low, possibly related to the magnitude of immune reinvigoration (35). Moreover, in a study of 233 patients enrolled in phase I trials of cytotoxic and molecularly targeted agents at Princess Margaret Cancer Centre, these three factors were found to be predictive of early mortality (21). The overlap seen between our prognostic variables and those of prior studies indicate that factors reflective of underlying disease biology and patient fitness remain central to the clinical trajectory and survival outcomes, despite evolving changes in anticancer treatment over the last decade.

The remaining seven variables analyzed did not demonstrate independent prognostic value in our population. LDH, NLR, and platelet count—laboratory parameters that are possible surrogates of tumor burden and inflammation—were statistically significant in univariate analysis, but not in MVA (Table 3). These factors have been observed to be prognostic in several phase I prognostic indices (20, 22, 23, 25, 26, 28, 30, 31, 34), and a high NLR is associated with adverse survival in various solid tumors (36). In our study, the number of patients with elevated platelet count was low (7%). Interactions and collinearity may have existed between these variables affecting the MVA outcomes. In keeping with multiple earlier studies (20, 21, 23, 24, 30, 34), we did not find age or the number of prior systemic therapies to be prognostic, supporting the notion that suitability for clinical trial participation should not be directed by these factors. Although prior exposure to multiple lines of therapy may be an indication of treatment refractoriness, it is also plausible that such patients have biologically more indolent disease and may be more likely to be recruited to early phase trials.

This study also provides contemporary insights on treatment outcomes of phase I oncology trials. Although treatment response rates in phase I clinical trials have been traditionally reported and oft-quoted as approximately 5% (26), a pooled review of all National Cancer Institute Cancer Therapy Evaluation Program-sponsored phase I clinical trials of cytotoxic agents and molecularly targeted agents between 1991 and 2002 involving almost 12 000 patients reported ORR of 11% (37). Response rates varied depending on the type of trial, with lower rates seen in first-in-human studies, and trials that included one or more approved anticancer agents resulted in higher response rates (37). A subsequent large European phase I series also reported response rates of approximately 10% (25). Survival has been inconsistently reported and widely variable in previous phase I series, with OS observed to be between 4 and 10 months (20–29, 31, 38, 39).

Efficacy and survival results from our development cohort and validation cohort B were markedly improved compared with reports in previously published phase I studies. ORR approached 20% and median OS exceeded 12 months. A significant proportion of patients remained well enough to receive further therapy after investigational treatment discontinuation, including subsequent clinical trials. The difference seen in the outcomes of these patients may be due to a combination of factors, such as durable treatment effect translating into greater survival gains, superior patient fitness perhaps related to earlier referrals in the treatment course, and improvements in supportive care. Of note, durable disease control is emerging as an important efficacy endpoint for IO agents owing to their differing biological activity. Stable disease at 6 months was observed in 14% of patients in the development cohort. In contrast, the treatment response rate (7%) and median OS (9.1 months) seen in validation cohort A were more consistent with previously published phase I series. These differences may be related to the enrichment of the development cohort for IO therapy-sensitive tumor types, such as melanoma and non–small cell lung cancer. Furthermore, patients in the development cohort were largely recruited prior to the approval of PD-1/PD-L1 targeted agents, and such agents were only available through clinical trials.

Phase I trials are generally considered to be safe, with reported toxic death rates consistently less than 1% (25, 37, 39). This is supported by our findings where no treatment-related death was seen in all three cohorts. Interestingly, 90DM was 15–20% in all three cohorts, similar to other phase I series (20, 21, 24–26). Although expected survival of greater than 90 days is a near universal inclusion criterion in phase I trials, a significant proportion of patients succumb to disease shortly after commencing treatment, likely due to rapid progression of disease, highlighting the limitations of prognostication for patients with advanced cancers, even in the hands of experienced phase I trialists. Nonetheless, the favorable safety and comparable efficacy outcomes suggest that phase I trials should be perceived as a valid therapeutic option rather than held in reserve after exhausting standard treatment options. This shift in practice is demonstrated by the large proportion (47%) of patients in the development cohort who received subsequent systemic therapies, including other phase I trial treatments. In an analysis from 2003 to 2006 of phase I participants at the Gustave Roussy Institute, 102 of 180 (57%) patients received at least one line of chemotherapy after trial completion (39).

Our study has a number of limitations. First, there was heterogeneity in the included IO treatments and trial designs. A wide range of tumor types were also included with differing susceptibility to IO therapy and natural disease courses. On the other hand, broad representation achieved via multi-institutional collaboration reflects the phase I IO population at large, making our results more generalizable. Second, some variables used in previously published prognostic scores were not assessed, such as thromboembolism or tumor type. To avoid overfitting, we limited the number of variables assessed to 1 per 10 death events. Third, caution must be used in applying the PM-IPI outside of phase I clinical trials, because phase I patients represent a select cohort of cancer patients with excellent performance status and optimal organ function.

The PM-IPI prognosticates for survival and is associated with treatment outcomes in phase I IO trials. Although patient selection should be individualized, an objective and reproducible prognostic tool such as the PM-IPI may assist in clinical decision-making for IO early phase trials and in turn help accelerate the development of IO therapies. To complement and strengthen the clinical model, analyses of archival tumor samples are underway, using established and emerging molecular techniques, including assessment of tumor-infiltrating lymphocytes and immune-related gene expression signatures to characterize the pretreatment tumor microenvironment and evaluate its clinical impact in the IO phase I setting.

## Notes

Affiliations of authors: Division of Medical Oncology and Hematology, Princess Margaret Cancer Centre, Toronto, Canada (DD, YK, AS, AMJ, LW, ARAR, NBL, ARH, MOB, LLS, PLB); Department of Medicine, University of Toronto, Toronto, Canada (DD, YK, AS, AMJ, ARAR, NBL, ARH, MOB, LLS, PLB); Department of Medical Oncology, Peter MacCallum Cancer Centre, Parkville, Melbourne, Australia (CG, BT, JD).

This study was approved by the Princess Margaret Cancer Centre Research Ethics Board (REB #15–9269-CE) and the Peter MacCallum Cancer Centre Human Research Ethics Committee (LNR/17/PMCC/274).

DD participated in study design and coordination, patient management, data collection and analysis, and drafting and revision of the manuscript. CG participated in study coordination, patient management, data collection and analysis, and manuscript revision. YK participated in data collection, patient management, and manuscript revision. BT participated in study coordination, patient management, and manuscript revision. AS, AMJ, ARAR, NBL, ARH, MOB, and JD participated in patient management and manuscript revision. LW provided statistical support and contributed to manuscript revision. LLS participated in study design and coordination, patient management, and manuscript revision. PLB participated in study design and coordination, patient management, and drafting and revision of the manuscript. All authors read and approved the final manuscript.

BT has been a speaker and advisor for Amgen, Astellas, BMS, and Janssen-Cilag and an advisor for Bayer, MSD, Novartis, Sanofi, Tolmar, and Ipsen and has received travel support from Amgen, Astella, Bayer, and Sanofi. AS has been a consultant for Merck (compensated), Bristol-Myers Squibb (compensated), Novartis (compensated), and Oncorus (compensated) and has received grant and research support (clinical trials) from Novartis, Bristol-Myers Squibb, Symphogen AstraZeneca/Medimmune, Merck, Bayer, Surface Oncology, Northern Biologics, and Janssen Oncology/Johnson & Johnson. ARAR declares honoraria from Boehringer Ingelheim; consulting and advisory role for Lilly, Merck, and Boehringer Ingelheim; and research funding from CASI Pharmaceuticals, Boehringer Ingelheim, Lilly, Novartis, Deciphera, Karyopharm Therapeutics, Pfizer, Roche/Genentech, Boston Biomedical, BMS, MedImmune, Amgen, GSK, Blueprint Medicines, Merck, Abbvie, and Adaptimmune. NBL declares no related conflicts of interest; unrelated conflicts of interest include consultancy for Canadian Agency for Drugs and Technologies in Health and honoraria for independent CME lectures for Astra Zeneca, BMS, Roche, and MSD. ARH declares advisory and consulting roles for Genentech/Roche, Merck, GSK, BMS, Novartis, Boston Biomedical, and Boehringer-Ingelheim. MOB has served on advisory boards for Merck, BMS, Novartis, Immunocore, GSK, EMD Serono, and Sanofi and receives research support from Merck and Takara and Bio. LLS declares consulting roles for Merck (compensated), Pfizer (compensated), Celgene (compensated), AstraZeneca/Medimmune (compensated), Morphosys (compensated), Roche (compensated), GeneSeeq (compensated), Loxo (compensated), Oncorus (compensated), and Symphogen (compensated) and grant and research support from (for clinical trials) from Novartis, BMS, Pfizer, Boehringer-Ingelheim, Regeneron, GSK, Roche/Genentech, Karyopharm, AstraZeneca/Medimmune, Merck, Celgene, Astellas, Bayer, AbbVie, Amgen, Symphogen, and Intensity Therapeutics, and LLS’ spouse is a stockholder in Agios. JD declares consulting /advisory roles for Bionomics, Lilly, Eisai, BeiGene, and Ignyta and research funding from Roche/Genentech, GlaxoSmithKline, Novartis, Bionomics, MedImmune, BeiGene, Lilly, and Bristol-Myers Squibb. PLB declares research funding (to institution) from BMS, Sanofi, AstraZeneca, Genentech/Roche, Servier, GSK, Novartis, SignalChem, PTC Therapeutics, Nektar, Merck, Seattle Genetics, Mersana, and Immunomedics. The remaining authors have no conflicts of interest.

We gratefully acknowledge all the patients and families who have participated in the clinical trials included in this article.

This work was presented in part at the 2016 and 2017 American Society of Clinical Oncology (ASCO) Annual Meetings.

## Supplementary Material

pkz071_Supplementary_Data
