# Supplementary material for: Survival in Early Phase Immuno-Oncology Trials: Development and Validation of a Prognostic Index
Source: JNCI Cancer Spectr. 2019 Sep 19;3(4):pkz071. doi: 10.1093/jncics/pkz071 (PMC7050022; doi:10.1093/jncics/pkz071)
Supplement: pkz071_Supplementary_Data [file pkz071_supplementary_data.docx]

**Supplementary Table 1. Clinical trial drug targets – Development Cohort**

|  | Drug Target | Number of Patients |
| --- | --- | --- |
| 1 | PD-1 | 80 |
| 2 | PD-1 | 34 |
| 3 | PD-L1 | 32 |
| 4 | OX40 | 10 |
| 5 | PD-1 + CTLA-4 or PD-1 + VEGF | 8 |
| 6 | PD-L1 | 8 |
| 7 | PD-L1 + CTLA-4 | 6 |
| 8 | PD-L1 + OX40 | 4 |
| 9 | PD-L1 | 3 |
| 10 | PD-L1 + CD40 | 2 |
| 11 | PD-1 + CTLA-4 | 2 |
| 12 | PD-1 | 2 |
| 13 | PD-1 + VEGF | 1 |
|  | Total | 192 |

**Supplementary Table 2. Clinical trial drug targets – Internal Validation Cohort**

|  | Drug target(s) | Number of Patients |
| --- | --- | --- |
| 1 | PD-1 | 35 |
| 2 | GITR +/- PD-1 | 22 |
| 3 | PD-1 | 19 |
| 4 | LAG-3 +/- PD-1 | 10 |
| 5 | OX40 + PD-L1 +/- VEGF | 9 |
| 6 | IDO + PD-1 | 8 |
| 7 | CSF-1R + PD-1 | 6 |
| 8 | VEGF + PD-1 | 6 |
| 9 | TIM-3 +/- PD-1 | 5 |
| 10 | PD-L1 | 5 |
| 11 | PD-L1 | 5 |
| 12 | PD-1 | 3 |
| 13 | PD-1 | 3 |
| 14 | OX40 | 2 |
| 15 | PD-L1, CTLA-4 or both | 2 |
| 16 | 4-1BB + PD-L1 | 2 |
| 17 | CD40 + PD-L1 | 2 |
| 18 | CD40 + Ang-2/VEGF-A bispecific antibody | 2 |
| 19 | CD73 + PD-1 | 1 |
| 20 | PD-1 + CTLA-4 | 1 |
| 21 | PD-L1 + CTLA-4 | 1 |
| 22 | TIGIT +/- PD-L1 | 1 |
| 23 | MEK + PD-L1 | 1 |
| 24 | ICOS +/- PD-1 | 1 |
|  | Total | 152 |

**Supplementary Table 3. Clinical trial drug targets – External Validation Cohort**

|  | Drug targets | Number of Patients |
| --- | --- | --- |
| 1 | PD-1 | 50 |
| 2 | PSMA/ CD3 bispecific antibody | 6 |
| 3 | ICOS +/- PD-1 | 5 |
| 4 | MEK + PD-L1 | 5 |
| 5 | CD73 + PD-L1 | 4 |
| 6 | PD-1/ CTLA-4 bispecific antibody | 4 |
| 7 | PD-1 + PD-L1 | 3 |
| 8 | TIGIT + PD-L1 | 3 |
|  | Total | 80 |

PD-1, programmed cell death protein-1; PD-L1, programmed cell death ligand-1; CTLA-4, cytotoxic T-lymphocyte-associated antigen-4; VEGF, vascular endothelial growth factor; GITR, glucocorticoid-induced TNFR-related protein; LAG-3, lymphocyte activation gene-3; IDO, indoleamine 2,3-dioxygenase; CSF-1R, colony stimulating factor 1 receptor; TIM-3, T-cell immunoglobulin and mucin domain-3; Ang-2, angiopoietin-2; TIGIT, T cell immunoreceptor with Ig and ITIM domains; MEK,  mitogen-activated protein kinase; ICOS, inducible co-stimulator; PSMA, prostate-specific membrane antigen.

**Supplementary Figure 1a. Kaplan Meier Curves for overall survival in Validation Cohort A based on the Princess Margaret Immuno-oncology Prognostic Index (PM-IPI)**

| PM-IPI | No. | Died | Median OS  (Weeks) | 95% CI |
| --- | --- | --- | --- | --- |
| 0 | 24 | 9 | 82.6 | 45.3 – not reached |
| 1 | 54 | 19 | 67.9 | 39.0 – not reached |
| 2 | 56 | 30 | 34.4 | 25.6 – 48.9 |
| 3 | 18 | 16 | 20.7 | 12.1 – 34.6 |

**Supplementary Figure 1b. Kaplan Meier Curves for progression-free survival in Validation Cohort A based on the Princess Margaret Immuno-oncology Prognostic Index (PM-IPI)**

| PM-IPI | No. | Events | Median PFS  (Weeks) | 95% CI |
| --- | --- | --- | --- | --- |
| 0 | 24 | 20 | 9.1 | 7.1 - 15.4 |
| 1 | 54 | 39 | 15.3 | 8.4 – 21.1 |
| 2 | 56 | 47 | 7.8 | 7.0 – 9.0 |
| 3 | 18 | 18 | 10.9 | 4.9 - 15.0 |

**Supplementary Figure 2a. Kaplan Meier Curves for overall survival in Validation Cohort B based on the Princess Margaret Immuno-oncology Prognostic Index (PM-IPI)**

| PM-IPI | No. | Died | Median OS  (Weeks) | 95% CI |
| --- | --- | --- | --- | --- |
| 0 | 21 | 3 | not reached | 89.9 – not reached |
| 1 | 34 | 14 | 79.0 | 51.3 – not reached |
| 2 | 23 | 17 | 22.9 | 14.9 – 43.4 |
| 3 | 2 | 2 | 62.6 | 22.9 – not reached |

**Supplementary Figure 2b. Kaplan Meier Curves for progression-free survival in Validation Cohort B based on the Princess Margaret Immuno-oncology Prognostic Index (PM-IPI)**

| PM-IPI | No. | Events | Median PFS  (Weeks) | 95% CI |
| --- | --- | --- | --- | --- |
| 0 | 21 | 13 | 66.6 | 8.3 – not reached |
| 1 | 34 | 26 | 15.7 | 8.0 – 35.1 |
| 2 | 23 | 22 | 7.6 | 5.1 – 10.0 |
| 3 | 2 | 2 | 14.7 | 14.4 – not reached |
